# Effects of Linoleic Acid on Gut-Derived *Bifidobacterium breve* DSM 20213: A Transcriptomic Approach

**DOI:** 10.3390/microorganisms7120710

**Published:** 2019-12-17

**Authors:** Alice Senizza, Maria Luisa Callegari, Biancamaria Senizza, Andrea Minuti, Gabriele Rocchetti, Lorenzo Morelli, Vania Patrone

**Affiliations:** 1 Department for Sustainable Food Process (DiSTAS), Università Cattolica del Sacro Cuore, via Emilia Parmense 84, 29122 Piacenza, Italy; alice.senizza@unicatt.it (A.S.); marialuisa.callegari@unicatt.it (M.L.C.); biancam.senizza@virgilio.it (B.S.); gabriele.rocchetti@unicatt.it (G.R.); lorenzo.morelli@unicatt.it (L.M.); 2 Biotechnology Research Centre (CRB), via Milano 24, 26100 Cremona, Italy; 3 Department of Animal Science, Food and Nutrition (DiANA), Università Cattolica del Sacro Cuore, via Emilia Parmense 84, 29122 Piacenza, Italy; andrea.minuti@unicatt.it; 4 Nutrigenomics and Proteomics Research Center (PRONUTRIGEN), Università Cattolica del Sacro Cuore, via Emilia Parmense 84, 29122 Piacenza, Italy

**Keywords:** linoleic acid, conjugated linoleic acid, *Bifidobacterium breve* DSM 20213, transcriptomic analysis, stress response

## Abstract

Bacterial production of conjugated linoleic acid (CLA) has recently received great attention because of the potential health benefits of this fatty acid. Linoleic acid (LA) can be converted to CLA by several microorganisms, including bifidobacteria, possibly as a detoxification mechanism to avoid the growth inhibition effect of LA. In the present in vitro study, we investigated the gene expression landscape of the intestinal strain *Bifidobacterium breve* DSM 20213 when exposed to LA. Transcriptomic analysis using RNA-seq revealed that LA induced a multifactorial stress response in the test strain, including upregulation of genes involved in iron uptake and downregulation of genes involved in sugar and oligopeptide transport. We also observed reduced transcription of genes involved in membrane and pili biosynthesis. The upregulation of iron uptake was not related to any putative ability of LA to chelate Fe^2+^, but was somewhat linked to stress response. Furthermore, we demonstrated that LA increased reactive oxygen species (ROS) production in bacterial cells, activating an oxidative stress response. This response was proved by thioredoxin reductase transcription, and was primarily evident among bacteria cultured in the absence of cysteine. This is the first report of the potential mechanisms involved in bacterial LA transport and stress response in *B. breve*.

## 1. Introduction

The omega−6 fatty acid linoleic acid (LA) is a structural component of cell membranes, as well as a precursor of several types of eicosanoids that are involved in important physiological processes. Essential fatty acids, such as LA, can only be obtained through diet, and current regulations mandate that infant formula must contain LA and α-linolenic acid (ALA) [1].

Conjugated linoleic acid (CLA) refers to a group of LA isomers that naturally occur in ruminant fats and dairy products, and that have been intensively investigated for their potential health promoting properties [2]. CLA stored in the fat tissue and milk of ruminants constitutes the main source of CLA in the human diet [3]. The isomers most abundantly found in nature are c9,t11-CLA, the intermediates produced during the biohydrogenation of linoleic acid to stearic acid (C18:0) by some microorganisms in the rumen [3]. CLA-producing bacteria in rumen have attracted a great deal of interest. Additionally, c9,t11-CLA is produced through the conversion of vaccenic acid (C18:1, t11) in the mammary gland [4]. A similar pathway has been reported in the human colon, where linoleic acid is converted to vaccenic acid via CLA and, finally, to stearic acid [5].

Numerous bacteria have been reported to metabolize LA into CLA. The ability to convert LA into CLA is strain-specific [2], and CLA producers can be divided into two groups based on the isomer: 9,11 and t10,c12-CLA. Many CLA producers are lactic acid bacteria—in particular, various species of lactobacilli. Within this group of bacteria, the putative linoleate isomerase is reportedly the key gene responsible for the bioconversion of LA into CLA [3], although the mechanism remains unclear.

Among bifidobacteria, which are considered normal commensals of the human gastrointestinal tract, some species exhibit the ability to convert LA into CLA. Raimondi et al. screened 34 *B. breve* strains, and identified *B. breve* WC0421 as the major CLA producer [6]. In another study, O’Connell et al. [7] used gas-liquid chromatography (GLC) to investigate the capacity of different bifidobacteria species to convert LA into CLA, and found that *B. breve* NCFB 2258 was the strain that best converted LA. Both reports elucidated a strain-specific ability to convert LA into CLA. In *B. breve*, the MCRA protein was identified as a carbon-oxygen lyase family protein that may play a role in catalyzing the first step of CLA production [8]. Conversely, O’Connell et al. [7] used an insertional mutation approach and found strong evidence that the *mcrA* gene was not involved in this process.

It is unclear why bacteria convert LA, but one of the best-supported hypotheses is that CLA production may be a detoxification mechanism adopted by the bacterial cells. Koppová and colleagues [9] have studied rumen bacteria, and reported that the lag phase length is proportional to the fatty acid concentration, and dependent on the LA concentration. Interestingly, a higher number of unsaturated double bounds is associated with a greater inhibitory effect on bacterial cell growth [9]. Moreover, Fontes et al. [10] reported that *B. breve* NCIMB 702258 grown in the presence of LA in semi-skimmed milk exhibited an altered cell membrane composition.

In the present study, we evaluated the in vitro response to LA exposure of the *B. breve* strain DSM 20213 (LMG 13208; ATCC 15700; NCTC 11815). This strain was isolated from the infant intestine, and can produce CLA [11]. We investigated the effects of LA on *B. breve* DSM 20213 using RNA-seq analysis to identify genes that could be involved in the response to LA exposure. Finally, we confirmed and verified our results using chemical and microbiological experiments.

## 2. Material and Methods

### 2.1. RNA Extraction and Sequencing

To explore the strain’s behavior in the presence of LA, we performed RNA-seq analysis in triplicate, comparing *B. breve* DSM 20213 grown in MRS-cys-LA (0.5 g/L) to *B. breve* DSM 20213 grown in MRS-cys. Bacterial cultures were grown in MRS-cys and MRS-cys-LA to an optical density of 0.7 (log phase) at 600 nm (OD_600_). Then 10 mL of culture was centrifuged at 10,000× *g* for 10 min, the supernatant was removed, and the cell pellet was immediately stored at −80 °C until use. Samples were processed for total RNA extraction following the acidic-phenol/guanidine-isothiocyanate protocol [12]. RNA was purified using the Direct-zol RNA Miniprep kit (Zymo Research, Irvine, CA, USA) following the manufacturer’s instructions. RNA quality check, library construction, and sequencing were performed at BGI (Shenzhen, China) using a BGISEQ−500. RNA-seq data have been submitted to Gene Expression Omnibus (GEO) under the accession number GSE139284.

Under our experimental conditions, we generated about 21.86 M reads per sample, and detected a total of 1887 genes. Prior to downstream analysis, we removed sequencing reads that were low-quality or adaptor-polluted, or that had a high content of unknown bases (N). After filtering the reads, the clean reads were mapped to the reference genome using HISAT [13]. The average proportion of reads mapped was 88.37%. The mapping results for each sample were highly uniform, such that the samples were comparable. Clean reads were progressively mapped to reference transcripts using Bowtie2 (http://bowtie-bio.sourceforge.net/bowtie2/index.shtml), and the gene expression level in each sample was measured using RSEM [14]. Based on the gene expression levels, we identified the differentially expressed genes (DEGs) between the samples. These DEGs were used to perform a Kyoto Encyclopedia of Genes and Genomes (KEGG) pathway classification and functional enrichment.

### 2.2. Real-Time Quantitative PCR Analysis

To confirm the gene expression profile obtained by RNA-seq, we performed real-time PCR (RT-qPCR) analysis. We reverse transcribed 1 µg RNA using the iScript™ Advanced cDNA Synthesis Kit (BioRad, Hercules, CA, USA) following the manufacturer’s directions, and diluted the cDNA 1:10 in nuclease-free water. Then q-PCR amplifications were performed using 5 μL of 1:10 diluted cDNA and the KAPA SYBR^®^ FAST qPCR Master Mix (2X) Kit (Kapa Biosystems, Wilmington, MA, USA) in a LightCycler^®^ 480 Instrument II (Roche Diagnostics, Indianapolis, IN, USA). Each sample was analyzed in triplicate. Ct values were normalized using the housekeeping gene *recF*, as previously described [15]. The utilized primers are reported in Table 1.

### 2.3. Chemicals, Strain, and Culture Conditions

Linoleic acid was purchased from Sigma-Aldrich (St. Louis, MO, USA). The *B. breve* DSM 20213 strain was acquired from the DSMZ collection (Braunzschweig, Germany), and was cultured in an anaerobic chamber (Don Whitley Scientific, Bingley, West Yorkshire, UK) at 37 °C using MRS broth (BD Difco™ Lactobacilli MRS broth, Thermo Fisher Scientific, Waltham, MA, USA) with the addition of 0.5 g/L L-cysteine-HCl (MRS-cys). MRS-cys-LA broth was prepared by adding 0.5 g/L LA to MRS-cys.

### 2.4. Growth of B. breve DSM 20213 at Different Cysteine Concentrations

*B. breve* DSM 20213 was grown overnight (18 h) under anaerobic conditions in MRS-cys. We then used 500 µL of the overnight culture to inoculate (1% *v*/*v*) 50 mL fresh MRS medium, with final cysteine concentrations of 0.5 g/L, 0.250 g/L, 0. 125 g/L, and 0 g/L. To test the protective effect of cysteine against LA, we prepared the previously described samples with and without the addition of 0.5 g/L LA in the medium. The strain grown in MRS-cys was used as the control. This experiment was conducted at 37 °C under anaerobic conditions. Using a spectrophotometer, we measured the OD_600_ hourly for 16 h.

### 2.5. Growth of B. breve DSM 20213 in Iron Salts-Supplemented Medium

The overnight culture of *B. breve* DSM 20213 was used to inoculate 50 mL of MRS-cys (1% *v*/*v*) supplemented with 50 mM ferric citrate, 50 mM iron sulfur, or a combination of both (1:1), to investigate the role of iron in LA metabolization. Samples were prepared with and without the addition of 0.5 g/L LA. The strain grown in MRS-cys was used as the control. A spectrophotometer was used to measure the OD_600_ hourly up to 15 h, and every 10 h thereafter.

### 2.6. Thiobarbituric Acid Reactive Substance (TBARS) Assay

We performed the TBARS assay to quantify the lipid peroxidation marker malondialdehyde (MDA), according to the method reported by Zhang and Huang [16], with minor modifications. Briefly, 10 mL of cell culture was centrifuged for 10 min at 10,000× *g*. The pellet was then mixed in a tube with 1 mL 0.1% trichloroacetic acid (TCA), and placed in an ultra-sonic bath at room temperature for 3 min to promote extraction. Next, the tube was centrifuged (10,000× *g* for 10 min at 4 °C) and the supernatant was transferred to a new tube. A 200 µL aliquot of supernatant was mixed well with 1 mL 20% TCA containing 0.5% TBA. This mixture was boiled at 95 °C for 15 min, quickly cooled on ice, centrifuged at 10,000× *g* for 5 min, and then the supernatant was transferred to a cuvette. Finally, we measured the absorbance at 532 nm using a PerkinElmer Lambda 12 spectrophotometer (PerkinElmer, Shelton, CT, USA). A molar extinction coefficient of 155 cm^−1^ mM^−1^ was used for MDA determination. The results were corrected for the experimental blank, and the MDA concentration was calculated as described by Lin et al. [17].

### 2.7. Assessment of the Ability of LA to Chelate Fe^2+^

We assessed the ability of LA to chelate Fe^2+^ using the colorimetric assay previously reported by Carter [18], with minor modifications. Briefly, 1.61 mL of each sample (MRS-cys) was diluted 1:30 (*v*/*v*) and then transferred into a 2-mL cuvette, with the addition of 150 µL 0.30 mmol/L FeSO_4_ and 0.5 g/L LA. After a 5-min reaction time, we added 230 µL 0.80 mmol/L ferrozine solution to each cuvette. In the control, the ferrozine solution was replaced by distilled water to correct for the unequal colors of the sample solutions. After 15 min, we read the absorbance at 562 nm using a PerkinElmer Lambda 12 spectrophotometer (PerkinElmer, Shelton, CT, USA). The final results were expressed as mg EDTA equivalents/L, according to Santos and co-authors [19].

### 2.8. Extraction of Cell-Associated Lipids and Gas Chromatography (GC) Analysis

*B. breve* DSM 20213 was grown in MRS-cys and MRS-cys-LA, and then 50 mL of culture was centrifuged at 8000 rpm for 10 min, and the pellet was dissolved in 1 mL PBS buffer and transferred into a glass tube. Total lipids were extracted using 9 mL of chloroform/methanol (2:1, *v*/*v*) containing 5 μg/mL 10,12-tricosadiynoic acid (Sigma-Aldrich, St. Louis, MO, USA) for use as an internal standard. Tubes were incubated at room temperature for 2 h, and then the lower phase containing the total lipids was transferred into a new tube and gently dried under nitrogen flow. Fatty acids were methylated by in situ transesterification with 0.5 N methanolic NaOH in methanol, followed by 14% boron trifluoride in methanol. After methylation, the lipid phase was extracted with 1 mL hexane.

Using an autosampler, the samples were injected into a gas chromatograph (model 7820A GC; Agilent Technologies, Santa Clara, CA, USA) equipped with a fused silica capillary column (CP-Sil 88, 100 m × 0.25 mm × 0.20 mm; Agilent J&W GC column) and a flame-ionization detector. A 25 to 1 split ratio was used for injection of 2 μL hexane containing methyl esters. The carrier gas was ultrapure helium, and inlet pressure was maintained at 33.35 psi. The injector temperature was maintained at 250 °C, and the detector temperature at 255 °C. The oven temperature was initially 70 °C (held for 4 min), was increased by 10 °C/min to 180 °C (held for 10 min), and was then increased by 4 °C/min to 240 °C (held for 25 min), with a final run time of 60 min. Peaks were identified using the Supelco 37 Component FAME Mix (Sigma-Aldrich, St. Louis, MO, USA). CLA conversion was calculated as previously reported [20].

## 3. Results

### 3.1. Gene Expression of B. breve DSM20213 in Response to LA Exposure

We used an RNA-seq approach to examine the physiological and metabolic status of the *B. breve* DSM 20213 strain following in vitro exposure to 0.5 g/L of LA. Gene expression was compared between the intestinal bacterium grown in MRS-cys-LA compared to in MRS-cys, to identify genes that were differentially expressed between the two conditions. Gene ontology (GO) classification and functional enrichment were performed, revealing that the differentially expressed genes could be classified into three ontology clusters: molecular biological function, cellular components, and biological process (Figures S1 and S2).

**Figure 1:**
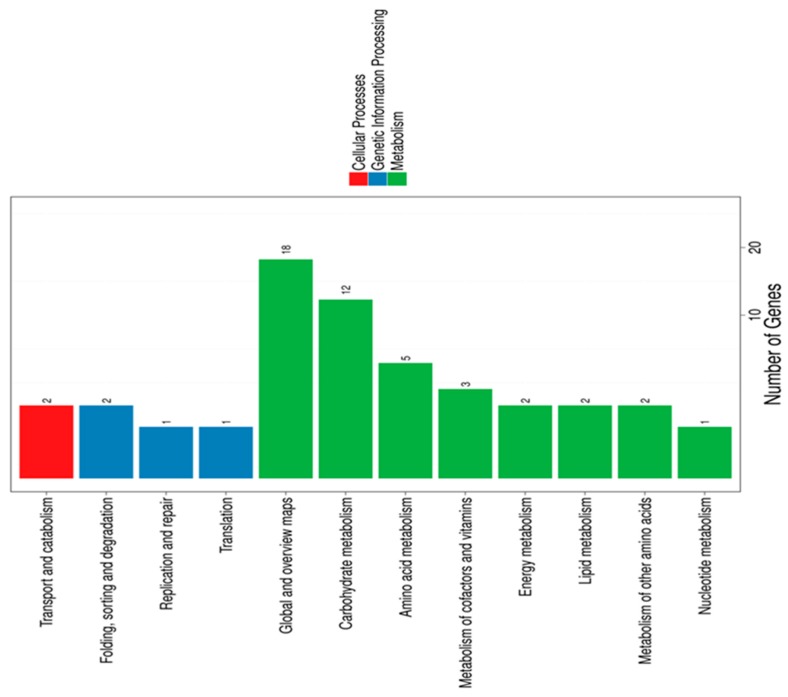
Kyoto Encyclopedia of Genes and Genomes (KEGG) pathway classification of differentially expressed genes. Genes were divided into three branches according to the biological pathways they participated in: cellular processes (red), genetic information processing (blue), and metabolism (green).

Overall, the highest numbers of DEGs were associated with “membrane,” “membrane parts,” and “catalytic activity”. We also performed KEGG pathway classification (Figure 1) and functional enrichment.

These analyses revealed that LA mainly impacted *B. breve* genes involved in carbohydrate and amino acid metabolism. Genes exhibiting less than a two-fold change were excluded from further analysis, leaving a total of 143 differentially regulated genes (53 upregulated and 90 downregulated) (Table S1). Gene annotation was achieved by searching the assigned locus tags in the NCBI Protein database for functionally characterized proteins displaying high similarity and/or mining locus tags associated with structural and functional annotations in the literature. In general, the most strongly upregulated genes were those involved in iron uptake systems, namely *bfeUO* and *sifABCDE* (Table 2).

The *bfeUO* system is responsible for ferric and ferrous import, and the *sifABCDE* system only for ferrous iron import. With regards to the *bfeUO* system, we found that LA exposure induced four-fold higher transcription of the BBRE_1003-encoded protein gene and three-fold higher transcription of the *BBRE_1002* gene, compared to control. In the *sifABCDE* cluster, LA exposure induced three-fold higher transcription of *BBRE_1006* and *BBRE_1007*, and two-fold upregulation of *BBRE_1004*, *BBRE_1005*, and *BBRE_1008* genes. The highest levels of induction (fold-change of 4) were observed for the *BBRE_1003* gene, a SAM-dependent methyltransferase (*BBRE_1612*), and an ABC transporter system gene (*BBRE_1753*). The function of *BBRE_1612* is not well-known, but this gene is reportedly upregulated under iron limitation conditions [21]. Table 2 lists the genes involved in iron uptake, regulators, and S-Fe cluster assembly proteins. The ferredoxin-NADP reductase gene (*BBRE_0908*) was also upregulated. These proteins harbor a prosthetic flavin cofactor, and catalyze reversible electron exchange with NADPH and either flavodoxin or ferrodoxin. Reduced ferredoxin can serve as an electron donor for many enzymes, playing roles in several systems, including oxidative stress [22].

The addition of LA to *B. breve* growth medium also impacted the expressions of two genes involved in sulfur amino-acid metabolism: upregulation of the cystathionine β lyase *metC* (*BBRE_1404*) and downregulation of a sulfatase family protein (BBRE_1630) (Table 2). Other differentially expressed genes were involved in membrane composition and pili formation. In terms of membrane modifications, LA apparently activated (fold-change of 2) the *BBRE_1618* gene that encodes a macrolide efflux protein, and *BBRE_1621* that encodes multidrug resistance protein B. These genes are strongly upregulated in the presence of cholic acid and oxgall [23], and represent the first microbial line of defense to protect the cell membrane from damaging compounds. Our results suggest that LA may be an inducer for this transport system, which could thus play a protective role in preserving membrane integrity.

LA exposure also influenced genes involved in regulation processes. The most highly expressed gene regulator was the WhiB-like protein WblE (BBRE_1505), which was originally isolated and identified by Averina et al. [24]. These iron-sulfur proteins have a redox-sensing function because of their four conserved cysteine residues. Averina et al. reported that this gene is upregulated under stress conditions, while the other two identified WhiB proteins exhibited no gene modulation. This line of evidence may implicate the WblE protein in sensing the external and intracellular redox state. Other upregulated genes included the ClgR regulator, which appears to be involved in solvent stress response [25], and *BBRE_0144*, which belongs to the xenobiotic response element family of transcriptional regulators (Table 2).

Overall, our results suggested that LA may have a negative regulatory effect on genes related to sugar transport, such as components of the PTS system and permease proteins of ABC transporter systems (Table S1). Of these, the PTS system IIC components were mostly downregulated. Such a reduction in sugar transport activity would impact carbon source utilization, consequently altering the production of ATP and important metabolic intermediates. Oligopeptide permease genes (*opp* gene cluster) were also downregulated—in particular, *oppB* (*BBRE_0967*) and *oppA* (*BBRE_0966*). Peptide transport systems play key roles in cell nutrition, as well as other processes, such as gene regulation expression, signaling, chemotaxis, conjugation, and competence development [26].

We also observed downregulated expression of genes involved in purine and pyrimidine metabolism. The ribonucleoside-diphosphate reductase system (*BBRE_0806*) exhibited two-fold lower expression in MRS-cys-LA than in MRS-cys. During cell division, this system is essential for converting ribonucleotides to deoxyribonucleotides, the precursors of DNA synthesis. Notably, downregulation of pyrimidine biosynthesis is a common strategy adopted by bifidobacteria to survive under stress conditions [27]. Our present findings regarding the DNA repair system suggest that LA does not induce DNA damage, based on the observed downregulation of DNA helicase II (*BBRE_1548*) (Table S1).

Interestingly, the presence of LA seemed to exert a negative effect on cell envelope. We observed reduced transcription of genes involved in lipid metabolism, including *BBRE_0137*, *BBRE_0138*, *BBRE_0139*, and *BBRE_0798*. We also observed downregulation of *BBRE_0139*, which is predicted to be a Type I multifunctional fatty acid synthase (fas). In *B. breve* UCC 2003 *fas* downregulation has been associated with reduced cell membrane integrity in response to bile [28]. This mechanism is often exploited by bacteria to survive under stress conditions [28,29]. Notably, LA reportedly modifies fatty acid composition in bifidobacteria cells [10].

Finally, RNA-seq analysis revealed that LA might also interfere with pili synthesis and thereby impact bacterial cell adhesion. We detected the downregulation of three predicted *Tad* genes. Bifidobacteria possess pili—adhesive and proteinaceous appendages that enable bacteria to adhere to host tissue [30]. Some of the genes encoding type IVb tight adherence (Tad) are expressed under several conditions, such as stress, or in the presence of other bacteria [31]. Our present results indicated that the *TadE* gene (*BBRE_0949*), which encodes one of the two pseudopilins and was identified in *B. breve* UCC2003 [30], exhibited three-fold lower expression in MRS-cys-LA than in MRS-cys. In contrast, *TadA* (*BBRE_0946*) and *TadC* (*BBRE_0948*) showed a fold-change of −1. TadC represents an integral membrane protein that enables the secretion of the pilus subunits, together with TadA. We also detected upregulation of *BBRE_0750*, with a fold-change of 2. This gene encodes the cell division protein Fic, which is involved in the catalysis of post-translational modification and regulation of bacterial filament. Members of the Fic protein family occur in taxonomically diverse bacteria and, interestingly, play a role in mediating bacterial responses to environmental stimuli. While we presently lack information regarding the role of the Fic protein family in modulating the shape of *B. breve* DSM 20213, optical microscope observation of this strain revealed reduced pleomorphism in MRS-cys-LA (Figure S4).

Among the sensor regulator genes, histidine kinase sensor (*BBRE_0275*) was downregulated with a fold-change of −3, suggesting that LA may be sensed by bacterial cells via this type of specific sensor/regulator mechanism. We also observed downregulation (fold-change of −2) of general stress response systems, such as the groEL and groES chaperonins.

To validate the transcriptomic data, we performed real-time quantitative PCR (RT-qPCR) analysis of five different genes (data not shown). The RT-qPCR results were in agreement with the results of transcriptomic analysis (r^2^ = 0.904).

### 3.2. LA Effect on Iron Metabolism

**Figure 2:**
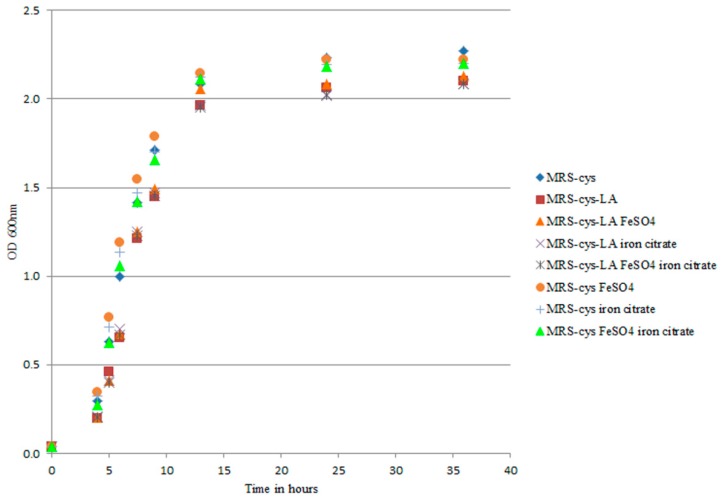
Growth curves of *B. breve* DSM 20213 grown in MRS-cys with iron sulfate, ferric citrate, and a 1:1 mix of both salts. The strain was exposed to 0.5 g/L linoleic acid (LA), and the optical density was measured at 600 nm. The growth of *B. breve* did not significantly differ among the tested experimental conditions (*p* > 0.05).

Transcriptomic analysis revealed that the addition of LA to *B. breve* growth medium had an impact on genes involved in iron uptake. In *Bifidobacterium longum* strain BBMN68, two different Fe-S cluster assembly proteins and an iron complex transport system are upregulated under oxidative stress conditions [32]. In that context, only the *suf* gene was induced and strain BBMN68 activated biosynthesis of the Fe-S cluster-including proteins to restore proper biochemical metabolism. Our present results demonstrated that the most highly upregulated genes were those belonging to *bfeUO* and *sifABCDE*. Moreover, the iron sulfur cluster-binding protein apbC (BBRE_1435) showed three-fold increased transcription with LA exposure compared to control. The higher gene expressions and the number of genes induced in the presence of LA in our study seemed to differ from those described in *B. longum* BBMN68 under oxidative conditions. The upregulation of several genes has also been described in *B. breve* UCC2003 under iron limitation conditions [21]. We conducted several experiments to exclude the involvement of LA in iron chelation. We first investigated the effect of iron salts on cell growth rate, with the rationale being to provide an extra source of iron to counteract an eventual iron deficiency in the medium. Under our experimental conditions, iron supplementation did not affect *B. breve* growth in MRS-cys (Figure 2).

We also examined the ability of LA to chelate iron, and our results excluded this hypothesis. The percentage of inhibition of complex formation did not differ between MRS-cys (90.744 mg EDTA eq/L) and MRS-cys-LA (90.969 mg EDTA eq/L). These results indicated that LA is not an iron chelator. Moreover, supplementation of the medium with iron did not improve the LA deconjugation, as there were no significant differences in the LA conversion (55.3 ± 3.3% in MRS-cys-LA; 58.5 ± 2.4% in MRS-cys-LA FeSO_4_; 54.9 ± 4.2% in MRS-cys-LA iron citrate; and 54 ± 3.5% in MRS-cys-LA FeSO_4_ iron citrate). Overall, our findings suggested that the induction of genes encoding iron uptake seemed to be correlated with a stress response because of the presence of LA in the medium.

### 3.3. LA Effect on ROS Production

It is well established that LA is involved in oxidative damage in mammalian cells and tissue; however, this relationship remains unclear in bacteria. Our present results showed that LA exposure modulated the expressions of a number of *B. breve* genes involved in oxidative stress pathways, prompting us to further investigate the putative role of LA in ROS production. We found that the relative intracellular MDA content significantly differed (*p* = 0.0117) between bacterial cells grown in MRS-cys-LA (16.61 ± 4.03%) versus those grown in MRS-cys (5.88 ± 1.27%).

**Figure 3:**
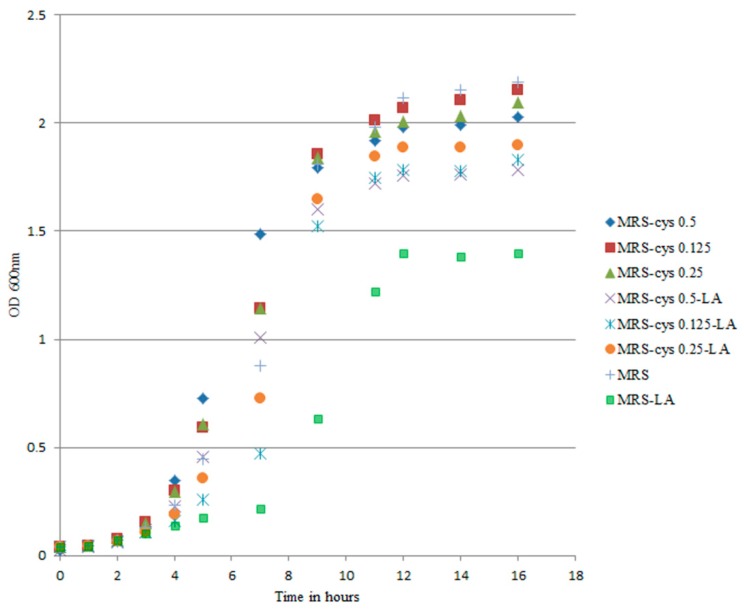
Growth curves of *B. breve* DSM 20213 grown in MRS-cys with different concentrations of cysteine. The linoleic acid (LA) concentration 0.5 g/L. Optical density was measured at 600 nm. The growth of *B. breve* in MRS-LA was significantly different from that observed in MRS (*p* < 0.05).

Cysteine is associated with a protective effect against oxygen during anaerobic bifidobacteria growth [33]. To explore the actual impact of cysteine on the oxidative status of *B. breve* under our experimental conditions, we reduced the cysteine concentration in the medium down to 0 g/L while maintaining an LA concentration of 0.5 g/L. The decreased cysteine content modified the trend of the growth curves, particularly in MRS-LA (Figure 3).

The part of the growth curve associated with the lag phase was most affected by changes in the medium. Changes in lag time are known to be a true consequence of stressful conditions [32]. We found that the lag phase was extended by approximately 8 h for *B. breve* growing in MRS-LA compared to in MRS (*p* < 0.05). Addition of LA did not affect the lag phase duration (approximately 5 h) of *B. breve* in MRS-cys. In MRS-LA, *B. breve* DSM 20213 showed a reduced growth rate, with the cell number reaching 7 log CFU/mL after 14 h of incubation, compared to 9 log CFU/mL for this strain in MRS. On the other hand, when the strain was cultivated in MRS without LA, supplementation with a lower cysteine concentration did not affect the lag phase duration, confirming that this amino acid played a limited role in the growth of this strain (Figure S3).

Our results led us to speculate that cysteine may play a putative protective role against the negative effects of LA. To check this hypothesis, we determined the MDA content in MRS and MRS-LA, and found that the MDA level was significantly higher in MRS-LA (34.80 ± 6.90%) than in MRS (8.21 ± 2.92%) (*p* = 0.0009). We also found that the relative MDA content significantly differed between bacteria grown in MRS-cys-LA (16.61 ± 4.03%) versus MRS-LA (34.80 ± 6.90%) (*p* = 0.0264). Overall, our findings support that LA may be implicated in triggering oxidative stress in *B. breve* DSM 20213.

The high levels of MDA in MRS and MRS-LA suggested that the strain grown in the absence of cysteine exhibited higher expression of genes involved in oxidative response. Thus, we performed q-PCR to determine the transcriptional level of the thioredoxin reductase gene. Thioredoxin reductase is considered to be one of the proteins responsible for maintaining a reduced intracellular environment. The expression of this gene was 1.64-fold higher in MRS-cys-LA than in MRS-cys, thus confirming the results of RNA-seq analysis. Conversely, this gene was upregulated by 2.46-fold in MRS-LA compared with in MRS. The highest gene upregulation (5.26-fold) was observed in MRS-LA compared with MRS-cys-LA. These results seem to suggest that the presence of LA activated an oxidative stress response, and that this negative effect is likely mitigated by cysteine.

## 4. Discussion

In the present study, we applied a transcriptomic approach to examine how LA exposure influenced the intestinal *B. breve* strain DSM 20213. In recent decades, host-microbiota interaction has been among the most explored research fields because of its importance in host health and disease. Microbial populations in the gut produce a number of active compounds, and thereby mediate the impact of nutrients on host physiology. Bifidobacteria represent the dominant colonizers of the large intestine of infants during the first weeks of life [34], and play key roles in the human gut because of their glycan-degrading abilities [35] and their capacity to utilize human milk oligosaccharides (HMOs) [36,37]. After lipids and lactose, HMOs are the most abundant solid component in breast milk, but they do not represent nutritional value for infants because they are resistant to pancreatic digestion [38]. Bifidobacteria exploit HMOs as a selective nutrient, to increase their competitiveness in the gut environment. Accordingly, bifidobacteria strains are utilized as probiotics for infant health and well-being [39]. Among the different species of the *Bifidobacterium* genus, *B. breve* has been formulated for clinical trials in pediatrics [40,41,42] and used as a supplement in infant formulas [43,44].

Essential fatty acids, such as LA, are important diet constituents, particularly during early infant development; however, they cannot be synthesized by the body. Thus, nutrition research has focused on the development of infant formula that is nutritionally balanced and similar to human milk [45]. To date, there is scarce in vivo evidence regarding the impact of unsaturated fatty acids on gut microbiota composition and function [46], and the available data have mainly been collected in animal models. De Weirdt et al. reported that increased linoleic acid in the cecum might affect the level of *Lactobacillus reuteri* in mice fed a diet high in polyunsaturated fat [47]. Their findings suggest that linoleic acid reaches the distal gut, and is metabolized by specific populations within the gut microbial community. Several investigations of the effects of LA on bacterial cultures reveal that this fatty acid is toxic to many bacteria, which cannot grow in the presence of LA [9,48]. It has also been reported that long-chain fatty acids with higher degrees of unsaturation exert stronger inhibitory effects on bacterial growth compared to fatty acids of the same chain length but with lower numbers of double bonds [49]. One primary effect of LA on bacterial cells could be the modification of membrane composition, as reported by Fontes et al. [10]. They found that bacteria cells grown in MRS-cys-LA exhibited significantly decreased levels of stearic, myristic, and lactobacillic acids, but not of palmitic and oleic acids. Boyaval et al. further demonstrated that LA increases membrane permeability, blocking the cell growth of *Propionibacterium freudenreichii* subsp. *shermanii*, and causing increased K^+^ efflux [50].

In the present study, we utilized RNA-seq methodology to assess the gene expression of *B. breve* DSM 20213 following exposure to LA, and to elucidate the in vitro effects of LA on the metabolic activities of the test organism. To our knowledge, this is the first study to show that LA can induce a multiple stress response in this microbial inhabitant of the infant gut.

Among the genes affected by LA, the most significant upregulation was detected for a cluster of genes related to the iron uptake system, which is often involved in bacterial responses to oxidative stress and adaptations to iron-limiting conditions [21,32]. *B. breve* DSM 20213 exhibited upregulation of both transcriptional units involved in iron uptake, although there was no differential expression of either thioredoxin reductase or glutaredoxin. In contrast, we observed upregulation of a ferredoxin NADP reductase gene encoding a protein with oxidoreductase activity, and the *WblE* gene that encodes a Fe-S protein involved in several stress responses, including the response to oxidative stress. To evaluate whether LA affected iron availability, we supplemented the growth medium of *B. breve* DSM 20213 with several different Fe sources and measured the bacterial growth rate. In this experiment, we also assessed the ability of LA to chelate Fe^2+^. Our results excluded that LA in MRS-cys was associated with any iron limitation effect, and excluded that LA had any direct involvement in bacterial CLA production as assessed by gas chromatography analysis.

Since our findings showed that LA exposure modulated several genes that are putatively involved in oxidative stress pathways, we explored the possibility that LA stimulated ROS production. We found that reactive oxygen species were significantly increased in *B. breve* cells grown in MRS-LA. The addition of cysteine to the medium seemed to protect bacterial cells against ROS-induced oxidative damage. Notably, we did not observe significant upregulation of genes encoding proteins with established roles in contrasting oxidative damages, possibly due to a protective action exerted by cysteine. Accordingly, thioredoxin reductase gene expression was higher in *B. breve* grown in the absence of cysteine compared to in the presence of cysteine, and this was the strongest effect recorded in MRS-LA.

Since LA exposure influenced genes and regulators involved in lipid metabolism, one could speculate that the cell membrane might be a target of LA action. Previous studies demonstrate that several kinds of stresses, including osmotic or acid stress, can prompt modifications in the bacterial cell membrane composition—particularly fatty acid chain length, saturation, and cyclopropanation—to decrease membrane permeability to oxidative free radicals and increase membrane resistance to lipid peroxidation [51]. We observed that LA induced downregulation of genes, such as the *TadE* gene, encoding type IVb tight adherence pili, which is in accordance with another transcriptional analysis showing reduced expression of these genes in response to stress conditions [52]. Pili are a key factor in bacterial adhesion and colonization of the host intestine [30]. As far as *B. breve* DSM 20213 is concerned, the involvement of pili in bacterial interaction with host tissue requires further clarification and, more specifically, the putative cell adhesion function of the *TadE* gene needs to be taken into consideration for future research. Despite the lack of evidence indicating that the cell filamentation protein fic is involved in morphological changes in bifidobacteria, RNA-seq analysis and observation with the optical microscope revealed a clear difference in *B. breve* DSM 20213 morphology—namely, a transition from a bifid shape to a rod shape.

Several species of bifidobacteria can convert LA into CLA, and the MCRA protein has been implicated in catalyzing the first step of CLA production in *B. breve* [8]. Our present results did not show any specific modulation of the *mcra* gene, suggesting that this enzyme was not involved in LA metabolization, which is in accordance with the findings of Raimondi et al. [6]. Along this line of evidence, O’Connell et al. [7] demonstrated that the MCRA protein of *B. breve* NCFB 2258 is an oleate hydratase that contributes to bifidobacterial solvent stress protection but not to CLA production. Indeed, Kishino et al. [53] proposed a multiple-step reaction mechanism for CLA production by the representative gut bacterium *Lactobacillus plantarum.* This pathway entails hydration, dehydration, and double-bond immigration and involves three different enzymes: CLA-HY, CLA-DH, and CLA-DC. Additionally, 10-hydroxy−12-octadecenoic acid has been identified as a key fatty acid intermediate of polyunsaturated fatty acid-saturation metabolism in *L. plantarum* [53]. Gao et al. [54] recently assessed the possibility that 10-hydroxy−12-octadecenoic acid, which is a product of MCRA activity in bifidobacteria, could represent an intermediate in bifidobacterial CLA generation. They demonstrated that *B. breve* can generate CLA using 10-hydroxy-*cis*-12 octadecenoic acid as a substrate. This aspect warrants further investigation, especially in light of recent findings indicating that *L. plantarum* multifunctional α-enolase can directly convert 10-hydroxy-12-*cis*-octadecenoic acid into c9,t11-CLA through dehydration and isomerization reactions [55]. Although we presently have only a limited understanding of how bifidobacteria can convert LA into CLA, multiple or alternative mechanisms may be involved, as shown for lactobacilli.

Some evidence suggests that the ability to convert LA into CLA is strain-specific [6], and that the conversion rate changes depending on the growth conditions and matrix [10]. A recent study demonstrated that *B. breve* Ncimb702258 could produce CLA in skimmed milk, but the LA conversion rate was lower than in synthetic medium. Despite the positive effects of CLA on human health, the new evidence that LA exerts a significant toxic effect towards *B. breve* bacterial cells warrants reconsideration of the extent of milk formula supplementation with this fatty acid.

The present study has several limitations—most importantly, that it is an in vitro study. Although such an approach is very useful to gain knowledge on the overall bacterial metabolic responses, it is not sufficient to predict the behavior of *B. breve* DSM 20213 in the complex and dynamic gut environment. The interplay between different microbial populations, as well as between bacteria and the host, could affect multiple metabolic processes occurring in the intestinal environment, including LA conversion.

In conclusion, in our present study, we examined the transcriptional landscape of *B. breve* DSM 20213 in response to LA. The evidence reported in this work confirmed the in vitro toxicity of LA on this bacterial strain. There remains a need for further investigations, combining metagenomics and metabolomics approaches, to understand the impact of LA on microbial community functions and interactions with the host in the human infant gut.

## Figures and Tables

**Table 1:** Oligonucleotide primers used for q-PCR analysis.

Gene (Locus Tag)	Primers Sequence (5′→3′)		Product Size (bp)
	Forward	Reverse	
*recF* (*BBRE_0834*)	GCACGCATCAATTCAGGAAC	GTCTTCAGGAGTAAACGAAAC	80
Cystathionine beta-lyase (*BBRE_1404*)	TGACCAATCCACTGCTCAAG	GCCAACACACCACCCAAAC	264
Permease protein of ABC transporter system (*BBRE_1006*)	CGCAGCAGATGAATGAGGAG	CCACCCAGTGATGATTGAGG	245
ATP-binding Mrp-like protein (*BBRE_1432*)	ACCAACCAGATCAACGGTGC	GCGGCAGAGAGAATCCATAG	294
Thioredoxin reductase (*BBRE_1224*)	CATTAACCATTGGCGGCCTG	GCTGACCACATCGGCAATGA	164
*Lex A* (*BBRE_0527*)	CGACTCCACATCGACGAAC	GCGTTCATGCGAATAAAGCC	133
Secreted protein probably involved in iron uptake (*BBRE_1003*)	CGCCAAGAAGGACGATTCAG	TTCACGGTCAGGTCAGGAAC	281
Ferredoxin--NADP reductase (*BBRE_0908*)	GTGACTGAATCTGAGAACACT	GATATCGGATGAGTAGACGC	77

**Table 2:** Expression changes in *B. breve* DSM 20213 following linoleic acid exposure.

Locus Tag	Protein’s Predicted Function	FC
BBRE_1003	Secreted protein, probably involved in iron uptake	4
BBRE_1002	Membrane-spanning protein with iron permease FTR1 family domain	3
BBRE_1006	Permease protein of ABC transporter system	3
BBRE_1007	ATP-binding protein of ABC transporter system	3
BBRE_1005	Permease protein of ABC transporter system	2
BBRE_1004	Membrane-spanning protein	2
BBRE_1008	Hypothetical protein	2
BBRE_1612	Hypothetical protein with methyl transferase domain	2
BBRE_1753	ATP-binding protein of ABC transporter system	4
BBRE_0908	Ferredoxin-NADP reductase	2
BBRE_1404	Cystathionine beta-lyase metC	3
BBRE_1630	Sulfatase family protein	−2
BBRE_1618	Macrolide-efflux protein, MFS member	1
BBRE_1621	Multidrug resistance protein B, MFS member, bile efflux induced upon bile salt exposure	2
BBRE_1505	WhiB protein, WblE	3
BBRE_0144	Transcriptional regulator	2
BBRE_0799	PTS system IIC component	−3
BBRE_0967	Oligopeptide transport system permease protein, oppB	−2
BBRE_0966	Oligopeptide-binding protein, oppA	−2
BBRE_0806	Ribonucleoside-diphosphate reductase alpha chain	−2
BBRE_0137	Acetyl-/propionyl-CoA carboxylase alpha chain	−2
BBRE_0138	Acetyl-/propionyl-CoA carboxylase beta chain	−2
BBRE_0139	Type I multifunctional fatty acid synthase	−2
BBRE_0798	Transcriptional regulator, GntR family	−2
BBRE_0896	Lysyl-cardiolipin synthase/Lysyl-transferase, mprF	2
BBRE_0949	TadE-like protein	−3
BBRE_0946	TadA-like protein	−1
BBRE_0948	TadC-like protein	−1
BBRE_0750	Cell division protein, Fic	2
BBRE_0275	Histidine kinase sensor of two-component system	−3
BBRE_0440	60-kDa chaperonin, GroEL	−2
BBRE_0182	10-kDa chaperonin, GroES	−2
BBRE_1435	ATP-binding Mrp-like protein	3

## Supplementary Materials

Supplementary materials can be found at https://www.mdpi.com/2076-2607/7/12/710/s1.
