# Supplementary material for: Effects of Linoleic Acid on Gut-Derived Bifidobacterium breve DSM 20213: A Transcriptomic Approach
Source: Microorganisms. 2019 Dec 17;7(12):710. doi: 10.3390/microorganisms7120710 (PMC6955684; doi:10.3390/microorganisms7120710)
Supplement: Supplementary file 1 [file microorganisms-07-00710-s001.pdf]

Effects of Linoleic Acid on Gut-Derived *Bifidobacterium breve* DSM 20213: A Transcriptomic Approach

Alice Senizza, Maria Luisa Callegari, Biancamaria Senizza, Andrea Minuti, Gabriele Rocchetti, Lorenzo Morelli and Vania Patrone

Table S1. Genes with a fold change (FC) of ≥2 in B. breve DSM 20213 following exposure to linoleic acid.

Locus tag	Protein and predicted functions	FC
	Catalytic activity	
BBRE_1751	ATP-binding protein of ABC transporter system	3
BBRE_1858	Hypothetical protein with DUF488 domain	3
BBRE_1404	Cystathionine beta-lyase, metC	3
BBRE_1752	ATP-binding protein of ABC transporter system	3
BBRE_1663	ATP-binding protein of ABC transporter system	3
BBRE_1435	ATP-binding Mrp-like protein	3
BBRE_0906	Trypsin-like serine protease	2
BBRE_0490	AAA family ATPase	2
BBRE_0126	Aspartate aminotransferase	2
	**Cofactor binding**	
BBRE_1008	Hypothetical protein	2
	**Integral component of membrane**	
BBRE_1753	ATP-binding protein of ABC transporter system	4
BBRE_1007	ATP-binding protein of ABC transporter system	3
BBRE_1002	Membrane-spanning protein with iron permease FTR1 family domain	3
BBRE_1613	Hypothetical protein	3
BBRE_1006	Permease protein of ABC transporter system	3
BBRE_1662	Permease protein of ABC transporter system	3
BBRE_0214	Membrane-spanning protein	3
BBRE_1661	Membrane-spanning protein	3
BBRE_1905	Hypothetical protein	3
BBRE_0844	Membrane-spanning protein	3
BBRE_0413	DedA family protein	3
BBRE_1655	Secreted protein	2
BBRE_1005	Permease protein of ABC transporter system	2
BBRE_1004	Membrane-spanning protein	2
BBRE_0473	Membrane-spanning protein	2
BBRE_0895	Esterase	2
BBRE_0356	Membrane-spanning protein	2
BBRE_0896	Lysyl-cardiolipin synthase/Lysyl-transferase, mprF	2
BBRE_1056	ATP-binding protein of ABC transporter system	2
BBRE_0149	Transporter MFS superfamily	2
BBRE_1895	ComE operon protein 3	2
BBRE_0687	Membrane-spanning protein	2
	**Regulator iron-sulfur cluster binding**	
BBRE_1505	WhiB protein, WblE	3
	**Transport**	
BBRE_1003	Secreted protein, probably involved in iron uptake	4
BBRE_1612	Hypothetical protein with methyl transferase domain	4
BBRE_1184	ATP-binding and permease modules of ABC transporter system	2
BBRE_1621	Multidrug resistance protein B, MFS member, bile efflux induced upon bile salt exposure	2

	**Oxidation-reduction process**	
BBRE_1111	Aspartate ammonia-lyase	2
BBRE_0908	Ferredoxin-NADP reductase	2
	**Metabolic process**	
BBRE_0631	Thiamin pyrophosphokinase	2
BBRE_1654	Pyrazinamidase/Nicotinamidase	2
BBRE_0414	AAA family ATPase	2
	**Transcriptional regulator**	
BBRE_1614	PEMK-like protein	2
BBRE_0144	Transcriptional regulator	2
BBRE_0680	Transcriptional regulator	2
BBRE_0529	Hypothetical protein with ATP cone domain	2
	**Cell division**	
BBRE_0750	Cell division protein, Fic	2
	**Others**	
BBRE_1859	Hypothetical protein	3
BBRE_1615	Hypothetical protein	2
BBRE_1620	Hypothetical protein	2
BBRE_1710	Hypothetical protein	3
BBRE_0681	Hypothetical protein	2
BBRE_1688	Hypothetical protein with DUF59 domain	2
**Locus tag**	**Protein and predicted functions**	**FC**
	**Integral component of membrane**	
BBRE_0275	Histidine kinase sensor of two-component system	−3
BBRE_1629	Glucose/fructose transport protein	−3
BBRE_0277	Membrane spanning protein	−3
BBRE_0276	Membrane spanning protein with PspC domain	−3
BBRE_0949	TadE-like protein	−3
BBRE_1084	Secreted protein with prokaryotic membrane lipoprotein lipid attachment site and Gram-positive cocci surface proteins, LPxTG motif profile	−2
BBRE_0011	Membrane-spanning protein	−2
BBRE_0727	Glycoside hydrolase	−2
BBRE_1140	Hypothetical protein	−2
BBRE_1313	Permease protein of ABC transporter system	−2
BBRE_0032	ATP-binding and permease protein of ABC multidrug transporter system	−2
BBRE_1101	Translation initiation inhibitor	−2
	**Carbohydrates transport**	
BBRE_0799	PTS system IIC component	−3
BBRE_0801	PTS system IIA component	−2
BBRE_0986	Phosphoenolpyruvate-protein phosphotransferase	−2
BBRE_0800	PTS system IIB component	−2
BBRE_0419	PTS system fructose-like IIB component	−2
BBRE_0263	PTS system fructose/glucose-specific IIABC component	−2
BBRE_0985	Phosphocarrier protein HPr	−2
BBRE_0797	Sugar ABC transporter ATP-binding protein	−2
BBRE_0195	Sugar-binding protein of ABC transporter system	−2
BBRE_0007	Sugar ABC transporter solute-binding protein	−2
	**Transport**	
BBRE_0648	ATP-binding protein of ABC transporter system	−3
BBRE_0306	Solute-binding protein of ABC transporter system (lactose)	−3
BBRE_0417	Nitrogen regulatory IIA (Enzyme IIA-NTR) (Phosphotransferase enzyme II A component)	−2
BBRE_0813	Potassium channel protein	−2
BBRE_0767	Permease protein of raffinose transport system	−2
BBRE_0196	Permease protein of ABC transporter system	−2
BBRE_1175	Permease protein of ABC transporter system	−2
BBRE_0270	Sugar ABC transporter permease protein	−2

BBRE_0931	Maltodextrin transport system permease protein, malC	−2
BBRE_0768	Permease protein of raffinose transport system	−2
BBRE_1185	Sugar ABC transporter solute-binding protein	−2
BBRE_0010	Sugar ABC transporter permease protein	−2
BBRE_0115	Transporter member of MFS, possible L-fucose permease	−2
BBRE_0967	Oligopeptide transport system permease protein, oppB	−2
BBRE_0269	Sugar ABC transporter permease protein	−2
BBRE_0197	Sugar ABC transporter permease protein	−2
BBRE_1372	ABC transporter permease	−2
BBRE_0171	Guanine-hypoxanthine permease	−2
BBRE_0966	Oligopeptide-binding protein, oppA	−2
	**Metabolic process**	
BBRE_1630	Sulfatase family protein	−2
BBRE_1312	Membrane-spanning protein, possible ABC-type permease	−2
BBRE_1543	Phosphate acetyltransferase	−2
BBRE_1000	Glutamyl-tRNA synthetase	−3
BBRE_0206	Zinc-dependent alcohol dehydrogenase family protein	−2
BBRE_0506	Mandelate racemase	−2
BBRE_0315	Lactaldehyde reductase	−2
BBRE_0965	Hydrolase	−2
BBRE_0547	Anhydro-N-acetylmuramyl-tripeptide amidase	−2
	**Lipid biosynthetic and metabolic process**	
BBRE_0138	Acetyl-/propionyl-CoA carboxylase beta chain	−2
BBRE_0137	Acetyl-/propionyl-CoA carboxylase alpha chain	−2
BBRE_0314	Cyclopropane-fatty-acyl-phospholipid synthase	−2
BBRE_0997	Carboxy-cis,cis-muconate	−2
BBRE_0507	Short-chain dehydrogenase	−2
	**Molecular function**	
BBRE_1001	DNA integrase	−3
BBRE_1548	DNA helicase II	−2
	**Carbohydrates metabolic process**	
BBRE_0929	Alpha-glucosidase	−2
BBRE_0480	Beta-fructofuranosidase	−2
BBRE_1628	Aldose 1-epimerase family protein	−2
BBRE_1922	N-acetylmannosamine-6-phosphate 2-epimerase	−2
BBRE_0271	Lacto-N-biose phorylase	−2
BBRE_0611	PfkB family carbohydrate kinase	−2
	**Branched chain amino-acid biosynthesis and metabolic process**	
BBRE_0088	3-isopropylmalate dehydratase small subunit	−2
BBRE_0087	3-isopropylmalate dehydratase large subunit	−2
	**Regulation of biological process**	
BBRE_0097	NADH oxidase H_2O-forming	−2
	**Regulator**	
BBRE_0798	Transcriptional regulator, GntR family	−2
BBRE_0716	WhiB family transcriptional regulator	−2
BBRE_0274	Response regulator of two-component system	−2
BBRE_0718	Macrolide ABC transporter ATP-binding protein	−2
BBRE_0770	Transcriptional repressor, ROK family	−2
BBRE_0017	Transcriptional regulator, MarR family	−2
BBRE_0715	WhiB family transcriptional regulator	−2
	**Protein folding**	
BBRE_0182	10-kDa chaperonin, GroES	−2
BBRE_0440	60-kDa chaperonin, GroEL	−2
	**DNA replication and cell division**	
BBRE_0806	Ribonucleoside-diphosphate reductase alpha chain	−2
BBRE_0717	DNA partition protein	−2

	**DNA recombination**	
BBRE_0738	Mobilization protein	−2
BBRE_1307	Transposase	−2
	**Oxidation-reduction process**	
BBRE_0139	Type I multifunctional fatty acid synthase	−2
	**Other**	
BBRE_0742	Hypothetical protein	−2
BBRE_1919	Appr-1-p processing protein	−2
BBRE_0743	Hypothetical protein	−3
BBRE_1100	Hypothetical protein	−2
BBRE_0089	Hypothetical protein	−2
BBRE_1335	Hypothetical protein	−2
BBRE_0744	Hypothetical protein	−2
BBRE_1129	Secreted protein with Gram-positive cocci surface proteins, LPxTG motif profile and Von Willebrand factor type A domain	−2
BBRE_1183	Hypothetical protein	−2
BBRE_0734	Hypothetical protein	−2
BBRE_1547	Toxin-antitoxin system antitoxin component, Xre family	−3

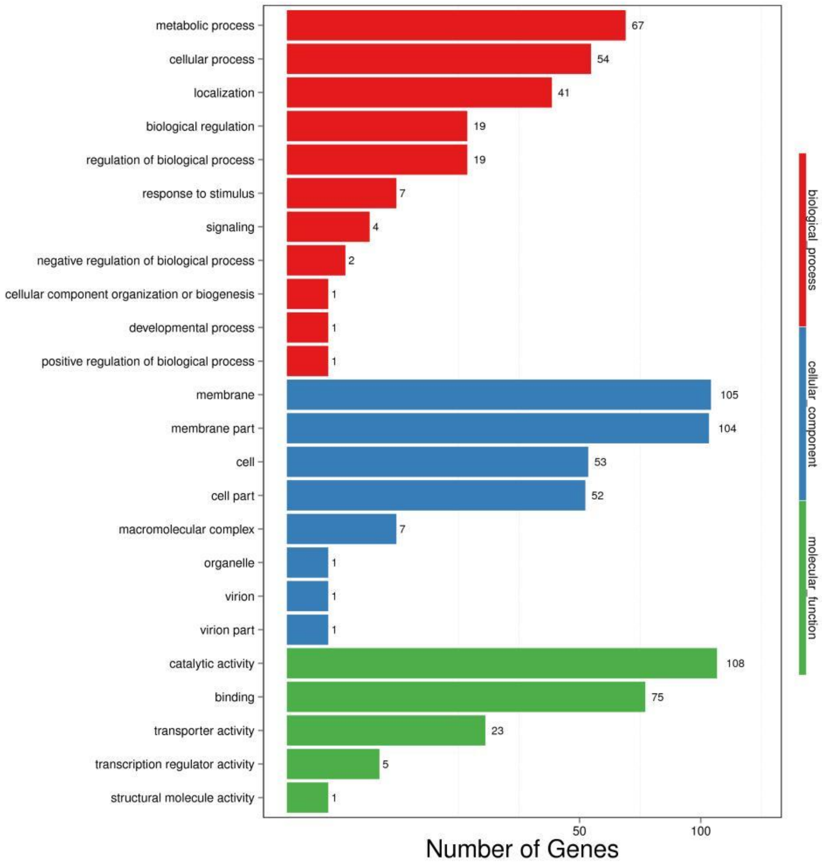

Figure S1. Gene Ontology (GO) functional classification of differentially expressed genes. The three main categories are reported: molecular biological function, cellular components, and biological processes.

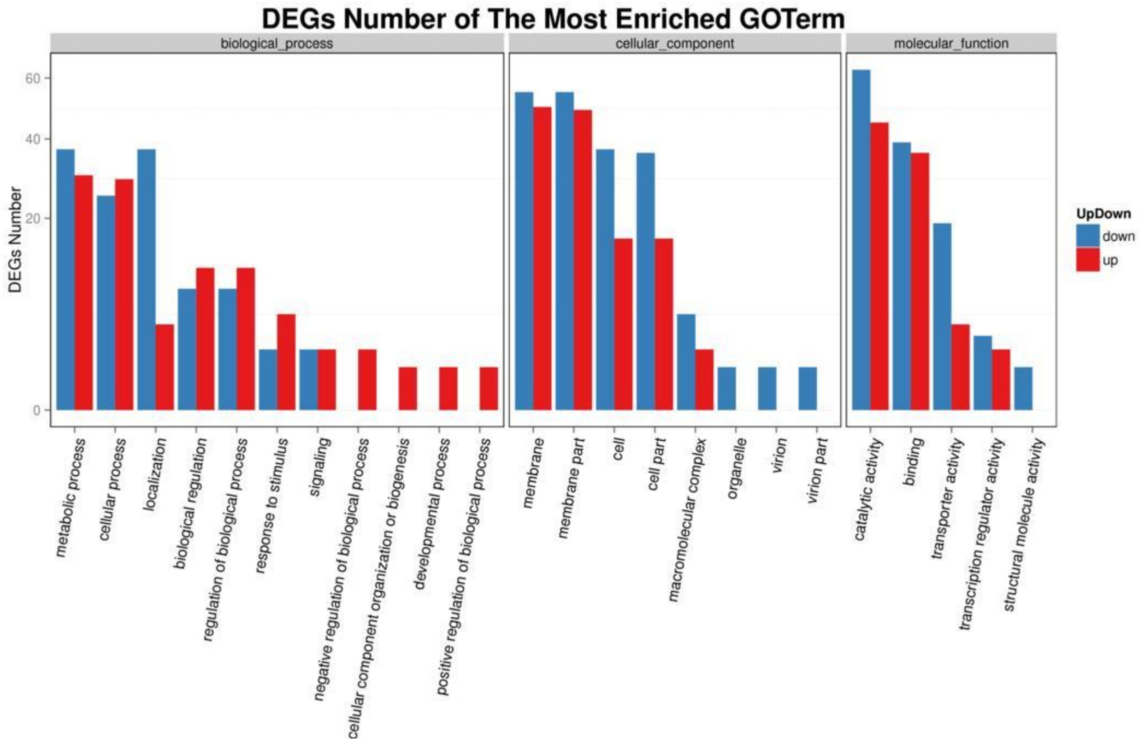

Figure S2. The Gene Ontology (GO) classification of upregulated genes (red) and downregulated genes (blue) in *B. breve* DSM 20213 grown in MRS-cys. compared to MRS-cys-LA.

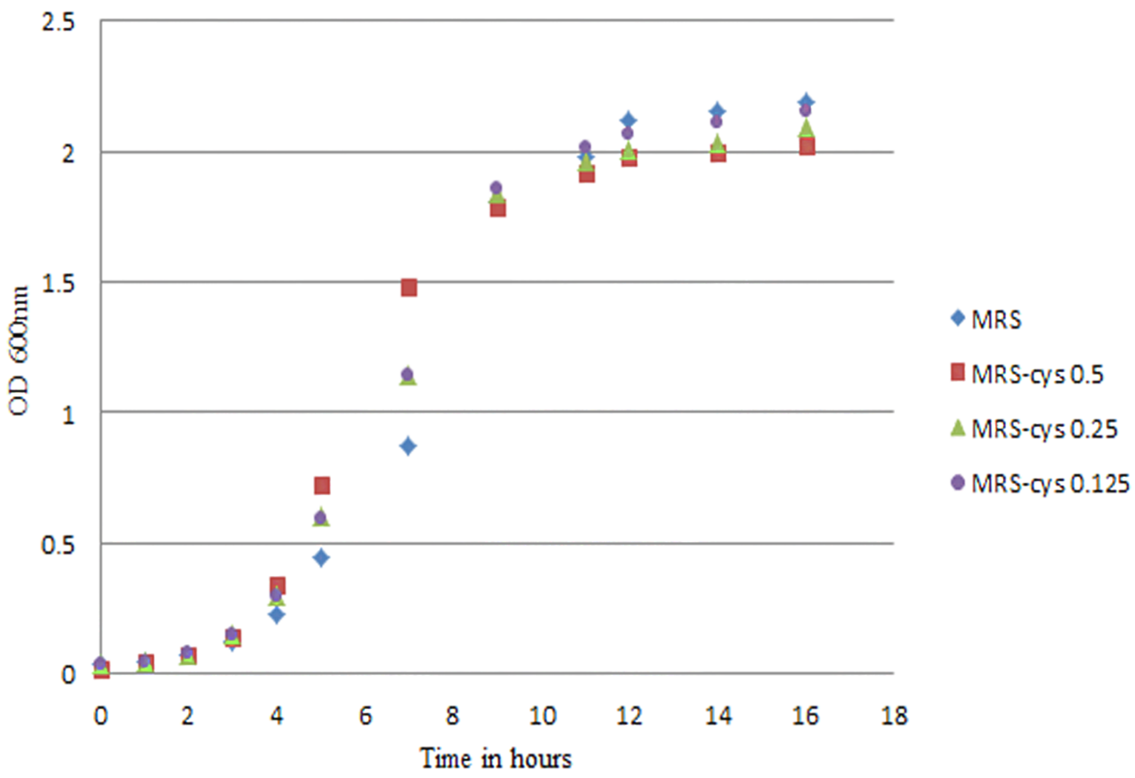

Figure S3. Growth curves of *B. breve* DSM 20213 grown in MRS-cys containing different concentrations of cysteine. Optical density was measured at 600 nm. No statistically significant differences ($p > 0.05$) were found between the growth curves of *B. breve* at different cysteine concentrations.

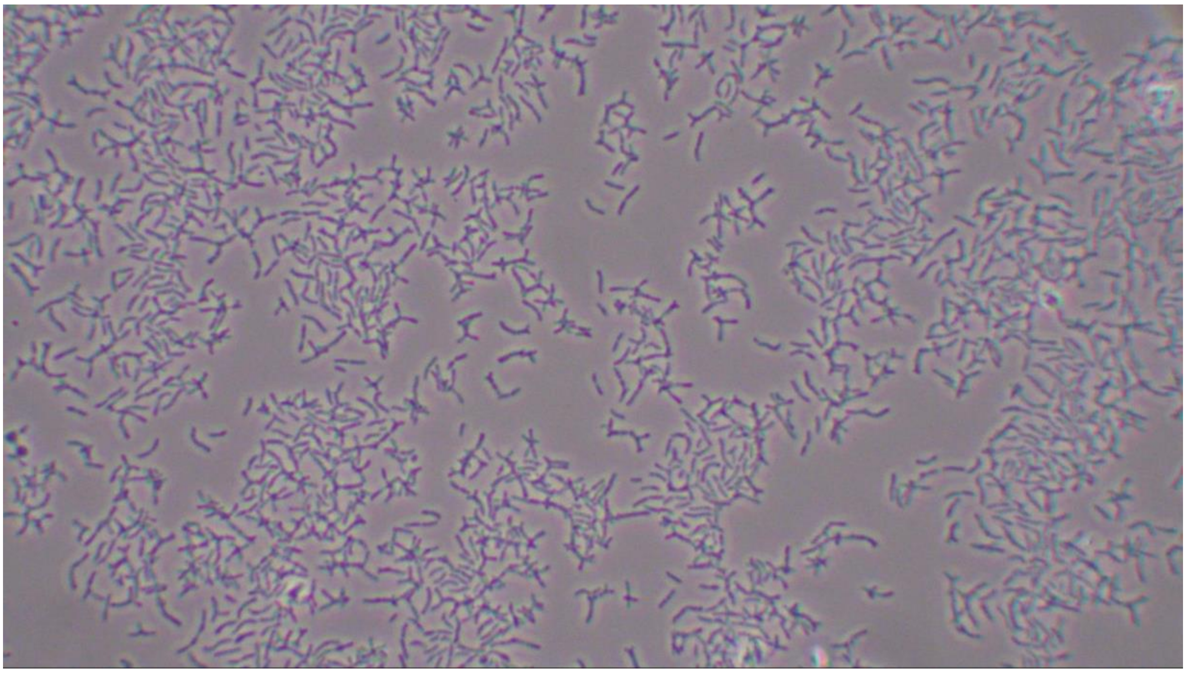

Figure S4a. Optical microscope observation of *B. breve* DSM 20213 grown in MRS-cys.

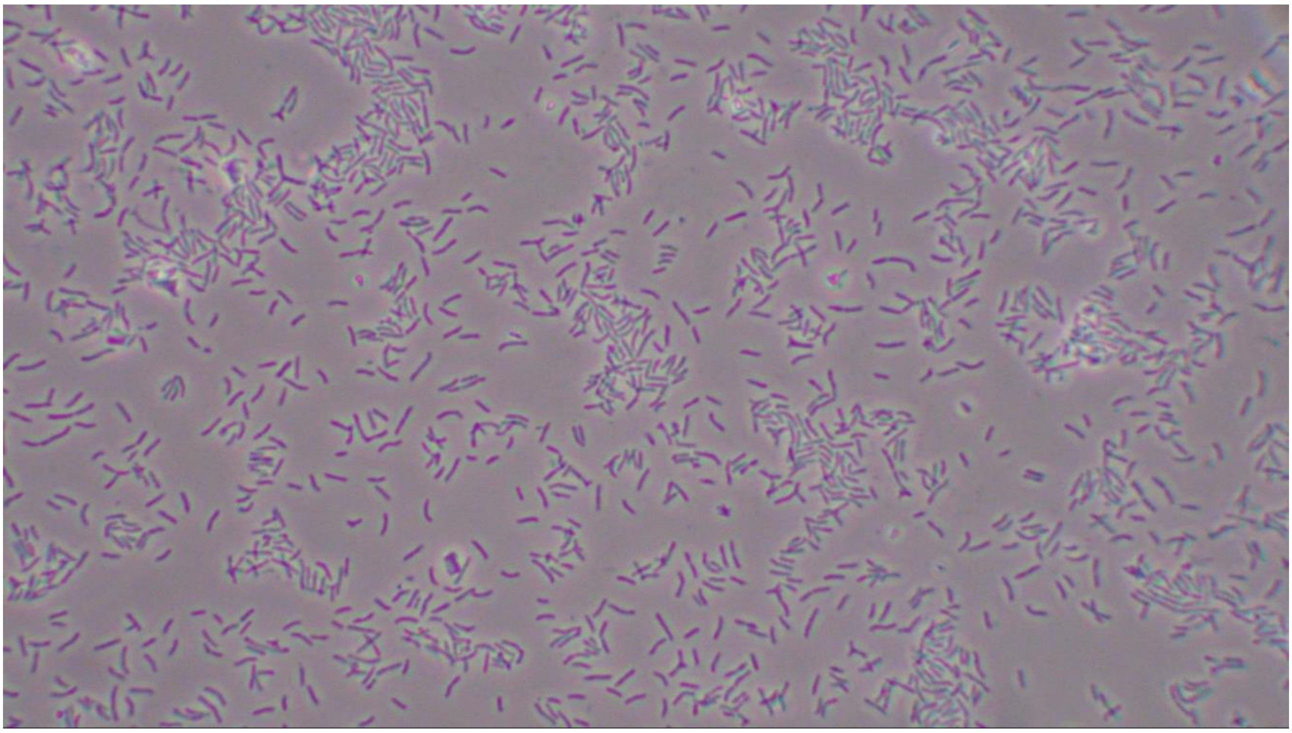

Figure S4b. Optical microscope observation of *B. breve* DSM 20213 grown in MRS-cys-LA.
